# Supplementary material for: Identification and classification of ion channels across the tree of life provide functional insights into understudied CALHM channels
Source: eLife. 2026 May 18;14:RP106134. doi: 10.7554/eLife.106134 (PMC13183375; doi:10.7554/eLife.106134)
Supplement: Figure 4—figure supplement 1—source data 1. [file elife-106134-fig4-figsupp1-data1.zip › Figure-supplementary-4_source-data_labeled.pdf]

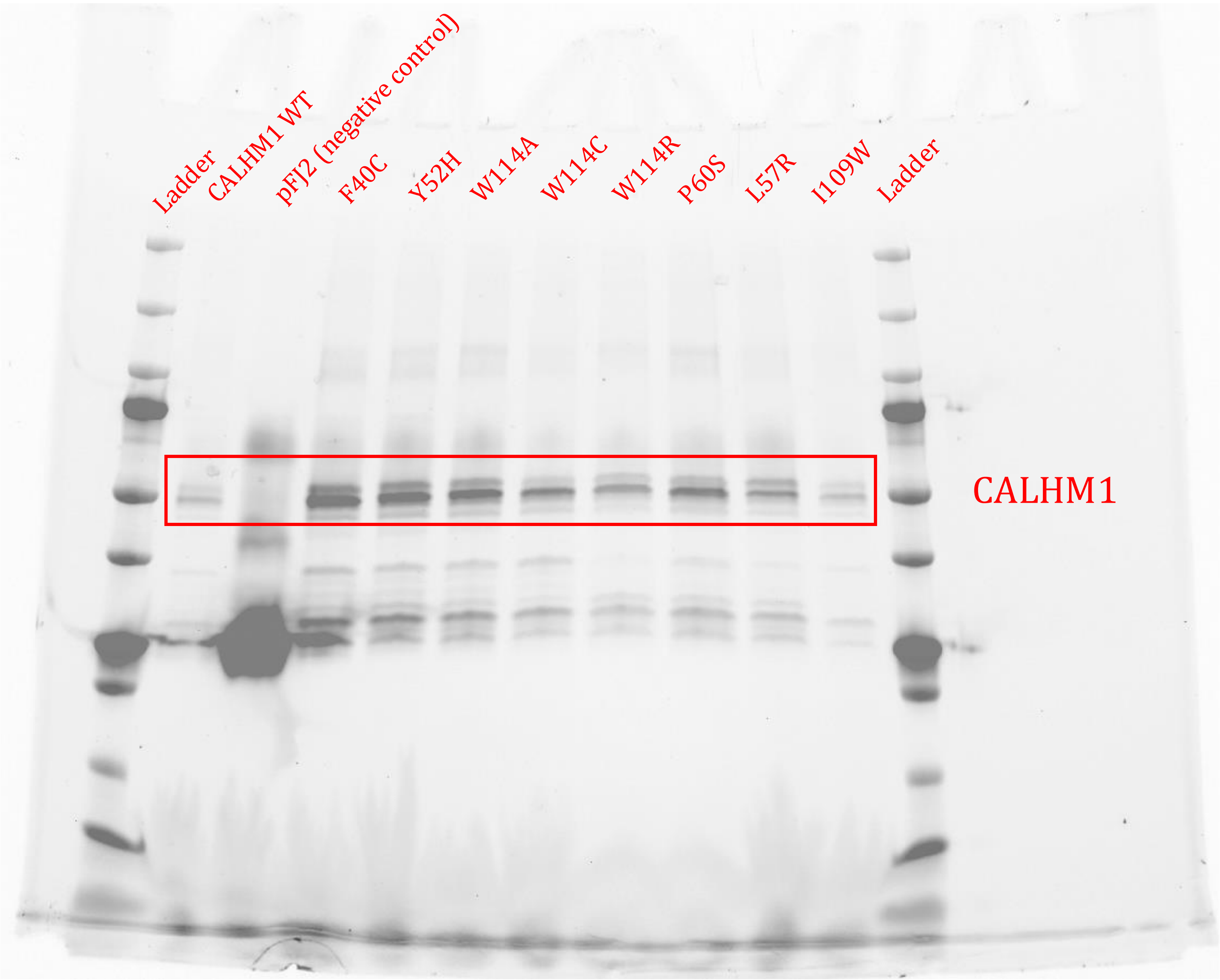

Ladder
CALHM1 WT
pFJ2 (negative control)
F40C
Y52H
W114A
W114C
W114R
P60S
L57R
I109W
Ladder
CALHM1

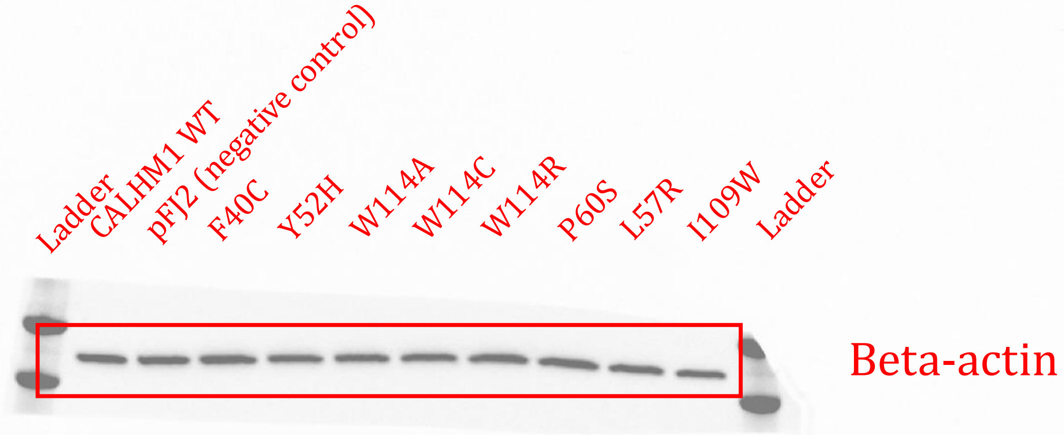
Ladder
CALHM1 WT
pFJ2 (negative control)
F40C
Y52H
W114A
W114C
W114R
P60S
L57R
I109W
Ladder
Beta-actin

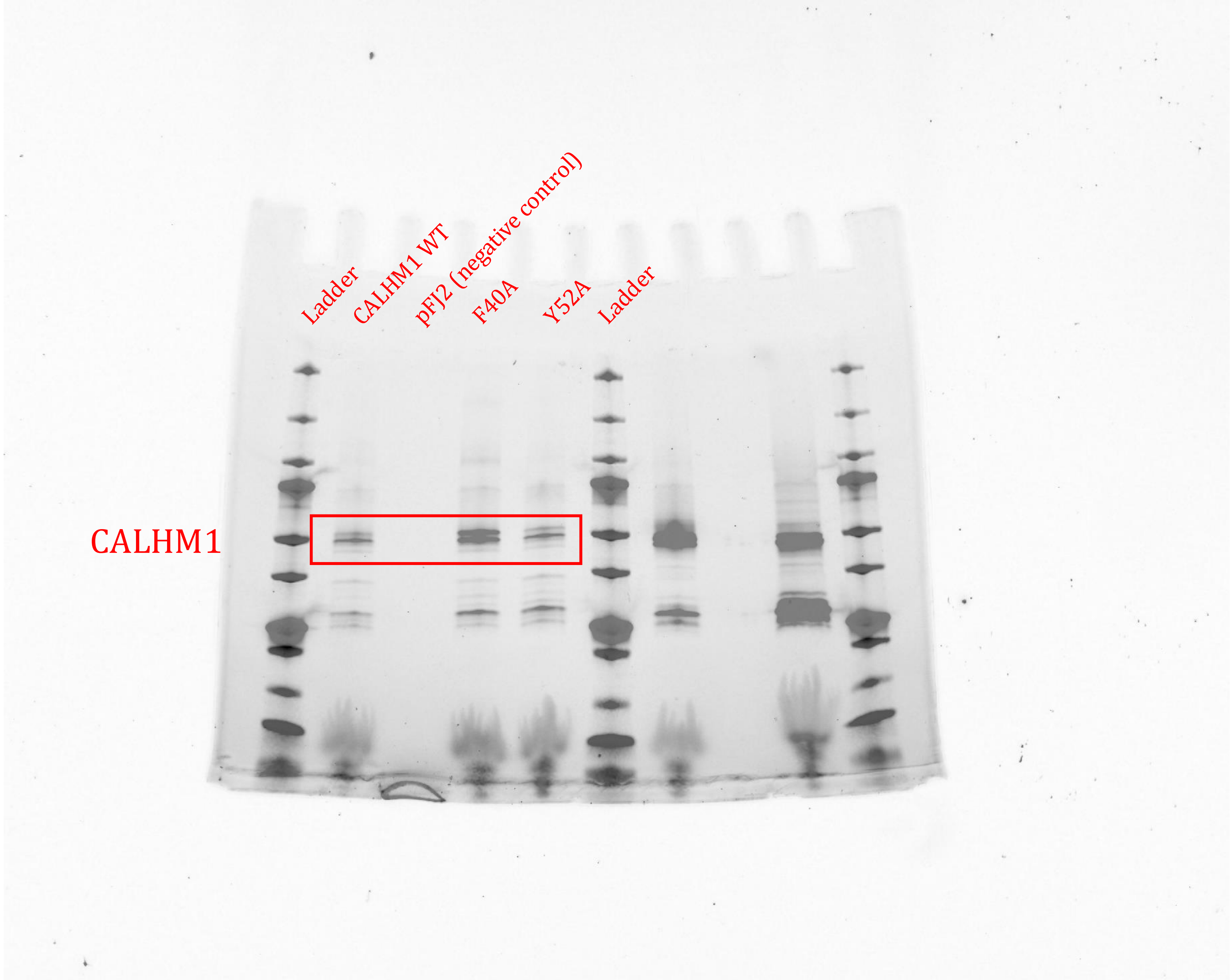
Ladder
CALHM1 WT
pFJ2 (negative control)
F40A
Y52A
Ladder
CALHM1

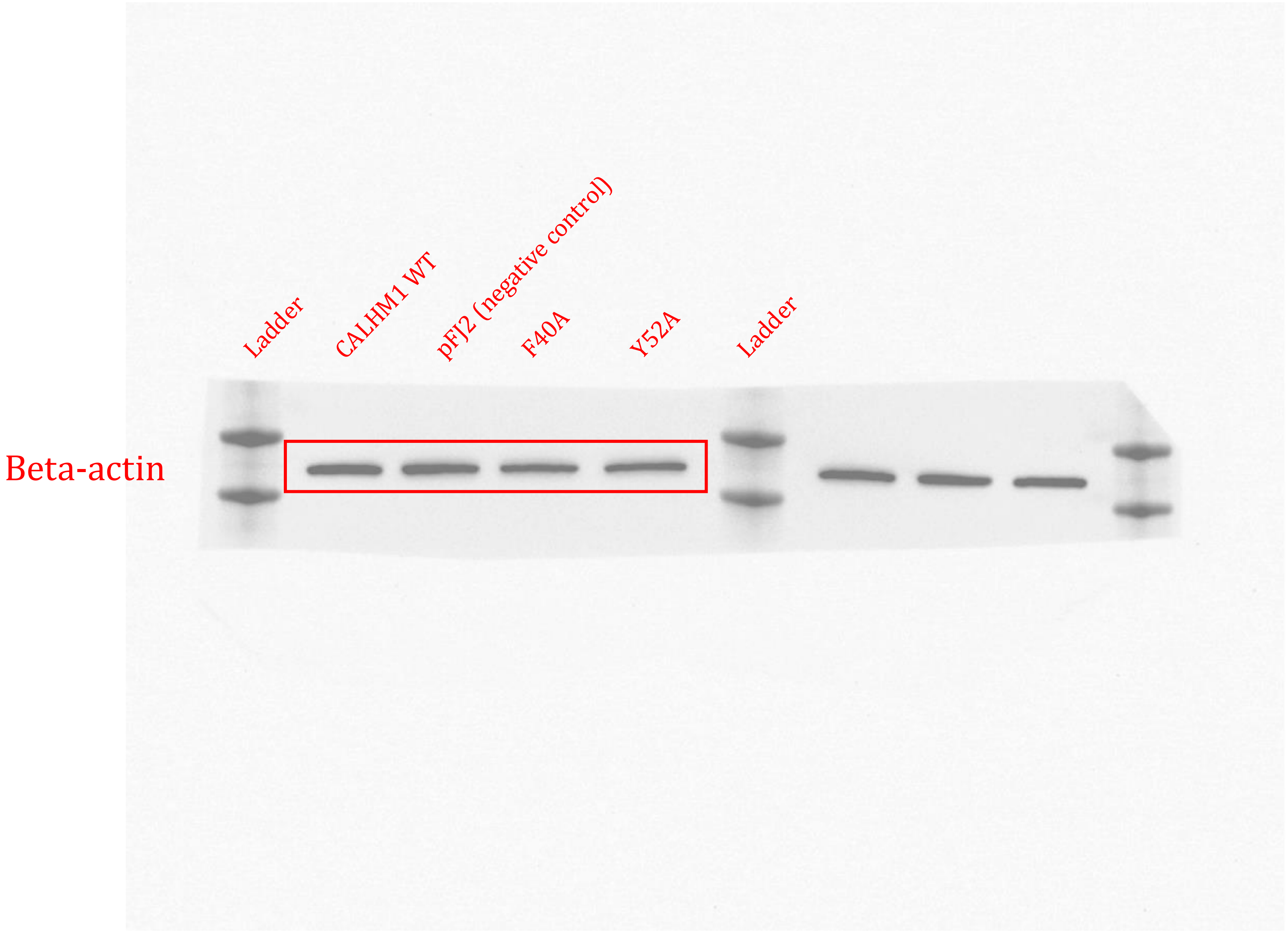
Ladder
CALHM1 WT
pFJ2 (negative control)
F40A
Y52A
Ladder
Beta-actin

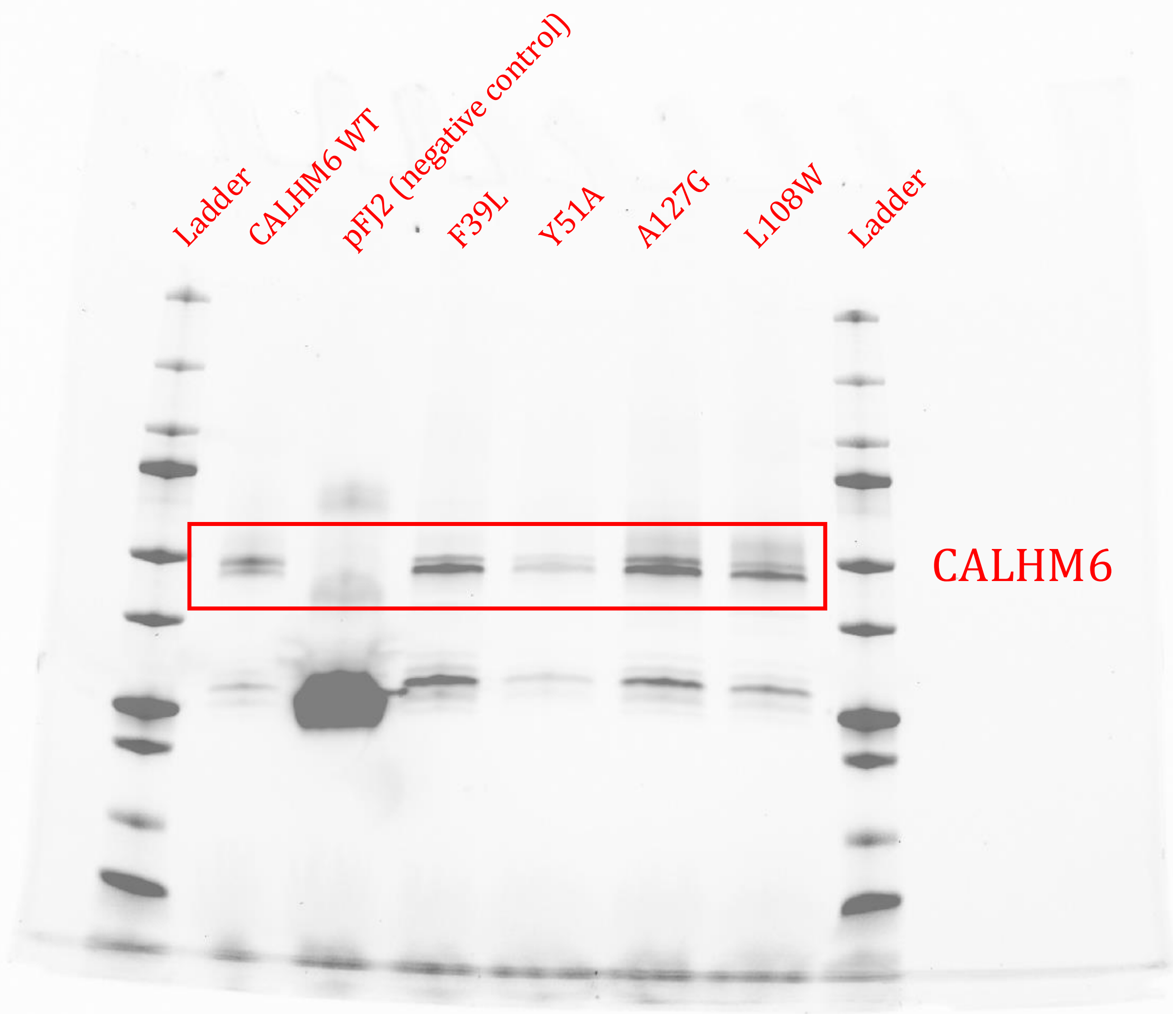
Ladder
CALHM6 WT
pFJ2 (negative control)
F39L
Y51A
A127G
L108W
Ladder
CALHM6

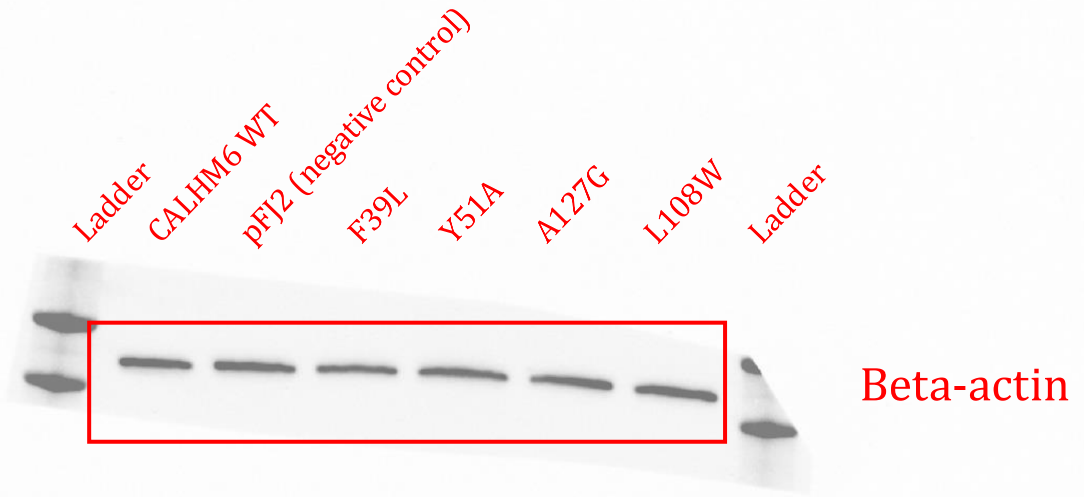
Ladder
CALHM6 WT
pFJ2 (negative control)
F39L
Y51A
A127G
L108W
Ladder
Beta-actin

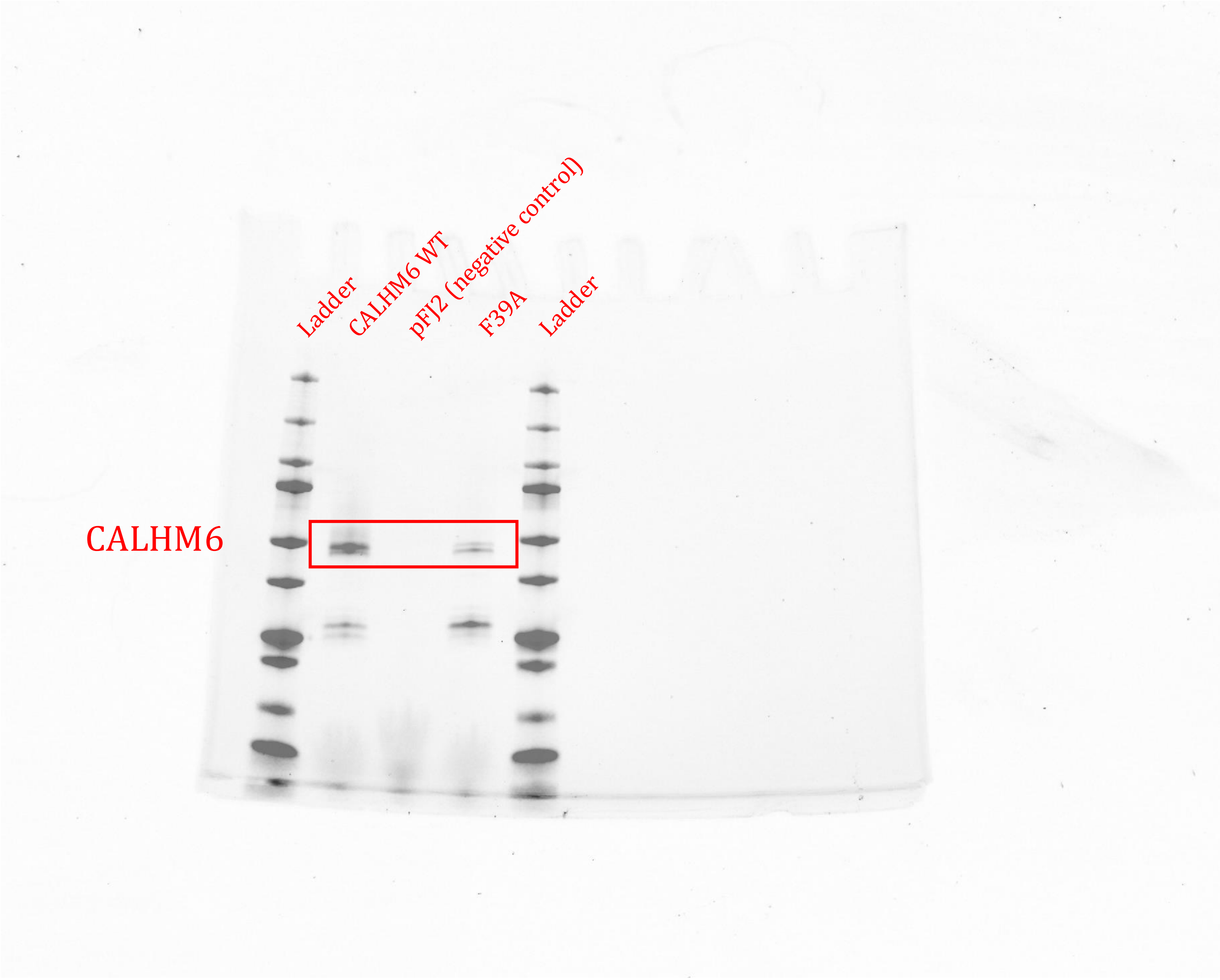
Ladder
CALHM6 WT
pFJ2 (negative control)
F39A
Ladder
CALHM6

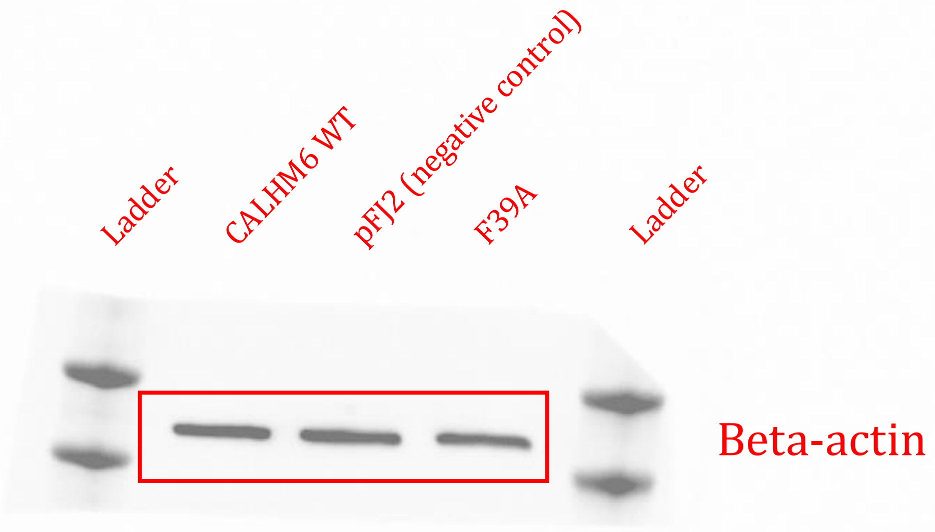
Ladder
CALHM6 WT
pFJ2 (negative control)
F39A
Ladder
Beta-actin

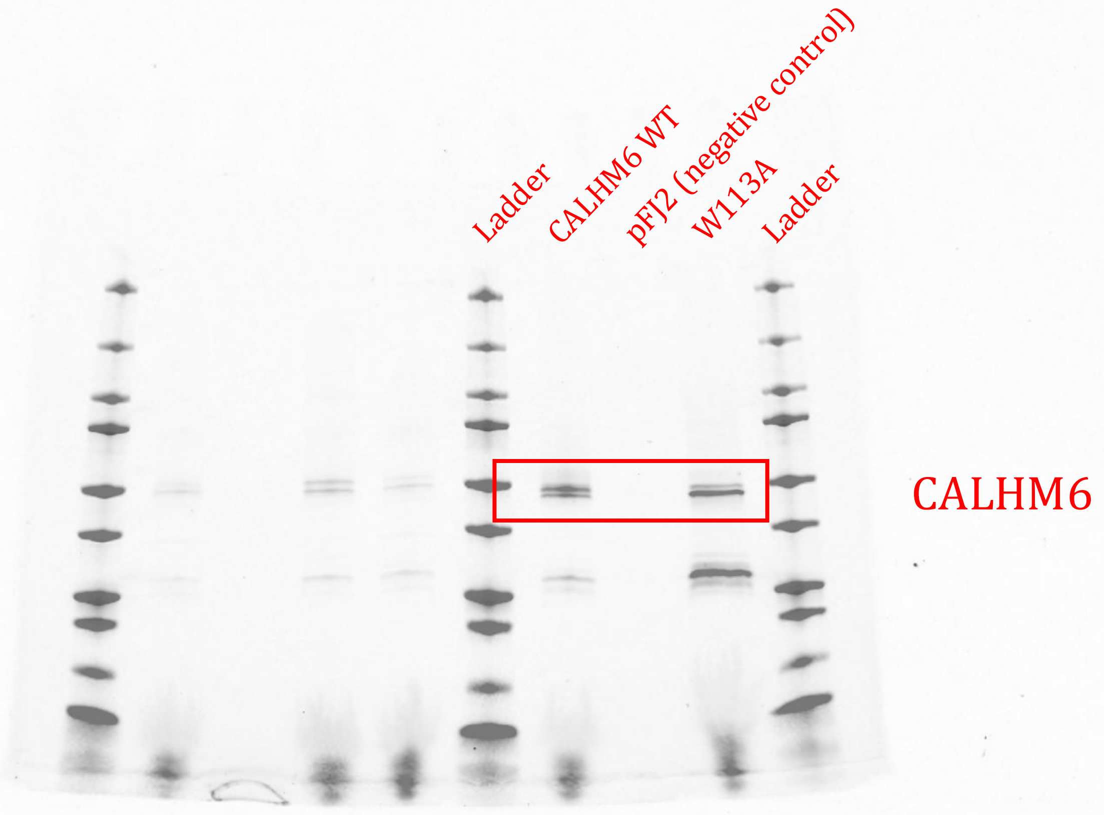
Ladder
CALHM6 WT
pFJ2 (negative control)
W113A
Ladder
CALHM6

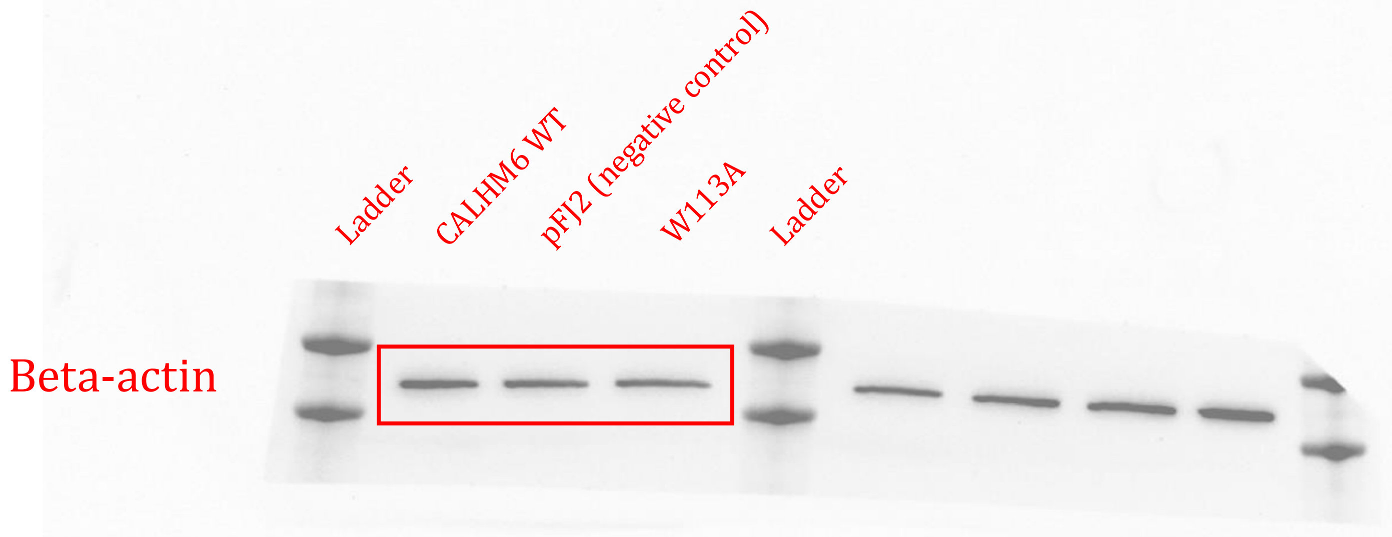
Ladder
CALHM6 WT
pFJ2 (negative control)
W113A
Ladder
Beta-actin

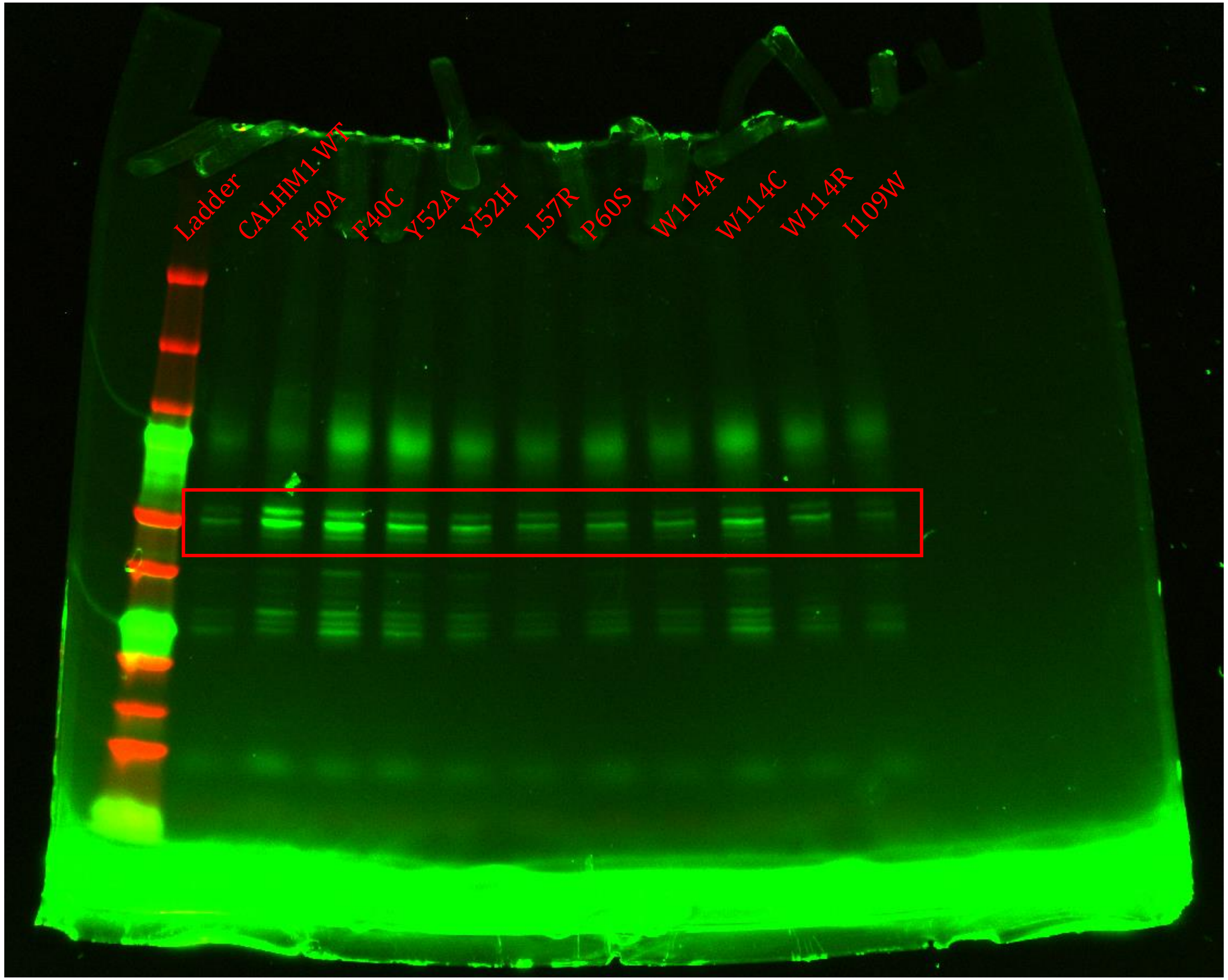
Ladder
CALHM1 WT
F40A
F40C
Y52A
Y52H
L57R
P60S
W114A
W114C
W114R
I109W

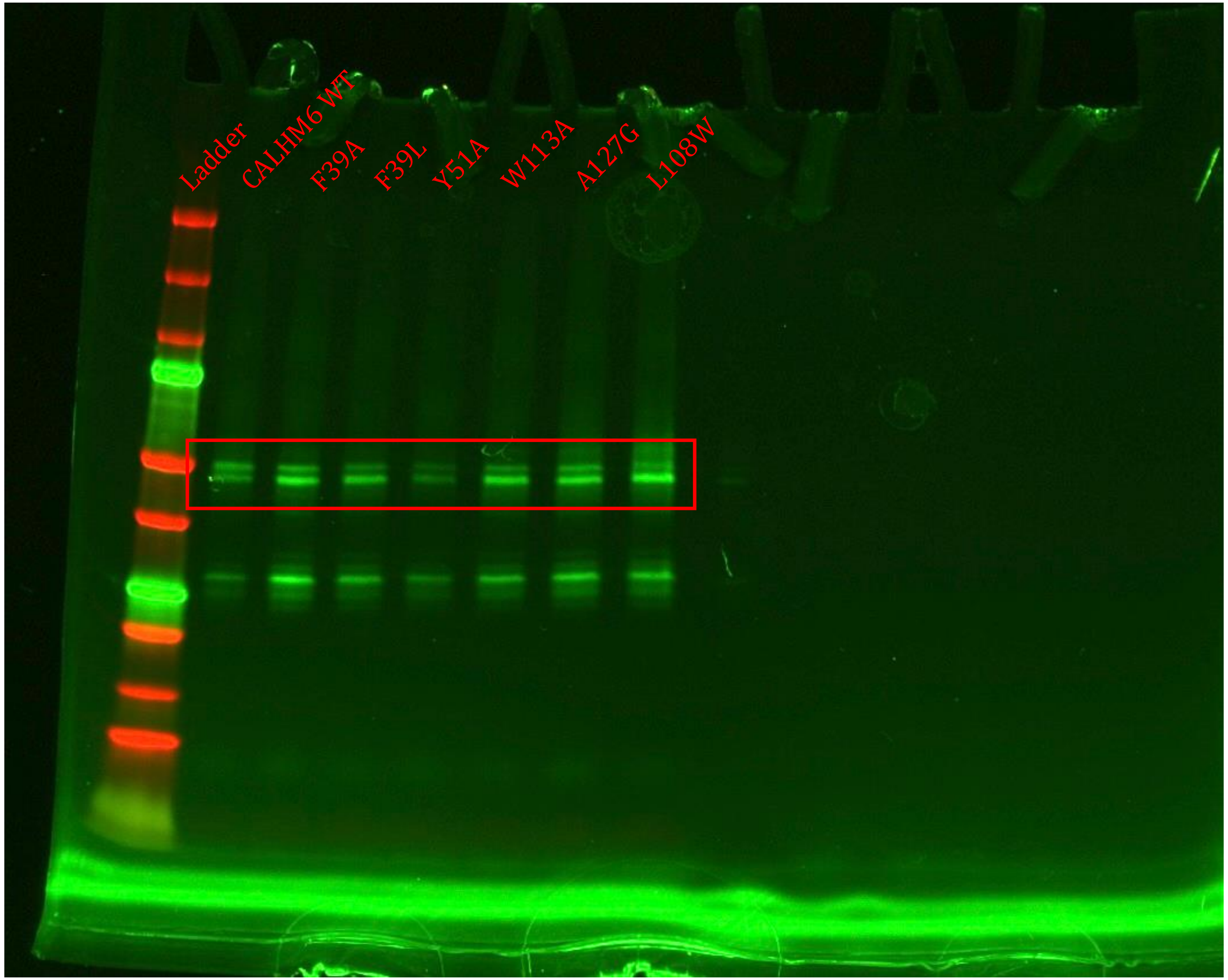
Ladder
CALHM6 WT
F39A
F39L
Y51A
W113A
A127G
L108W
