# Supplementary figures and images for: Identification and classification of ion channels across the tree of life provide functional insights into understudied CALHM channels
Source: eLife. 2026 May 18;14:RP106134. doi: 10.7554/eLife.106134 (PMC13183375; doi:10.7554/eLife.106134)

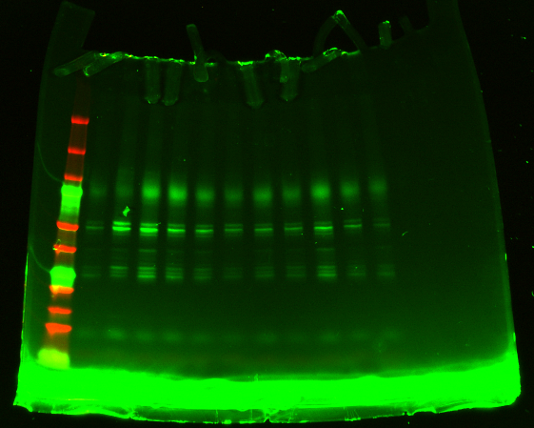

Supplement: Figure 4—figure supplement 1—source data 2. [file elife-106134-fig4-figsupp1-data2.zip › Figure-supplementary-4_source-data_raw/CALHM1_biotinylation_gel.png]

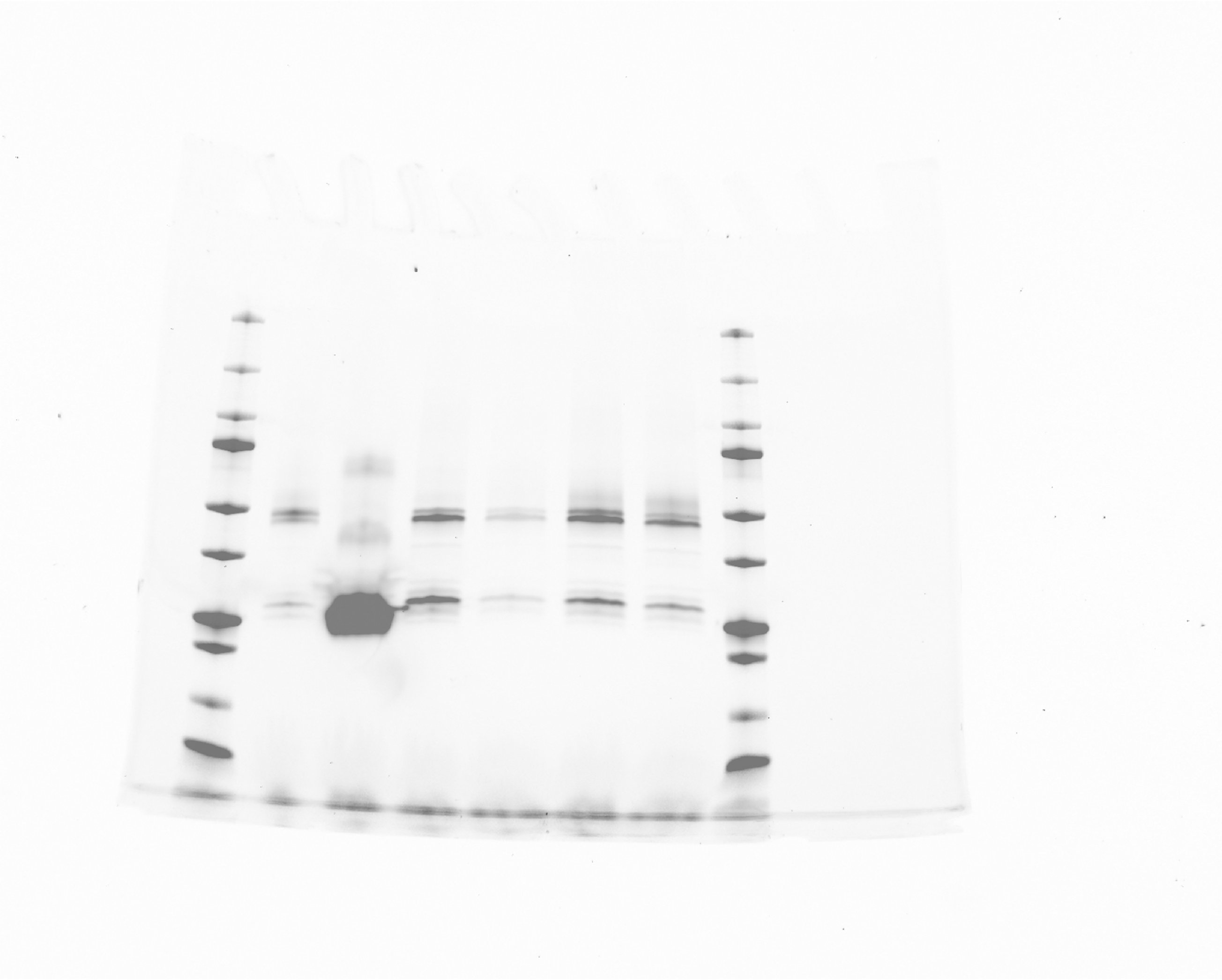

Supplement: Figure 4—figure supplement 1—source data 2. [file elife-106134-fig4-figsupp1-data2.zip › Figure-supplementary-4_source-data_raw/CALHM6_signal_gel1.png]

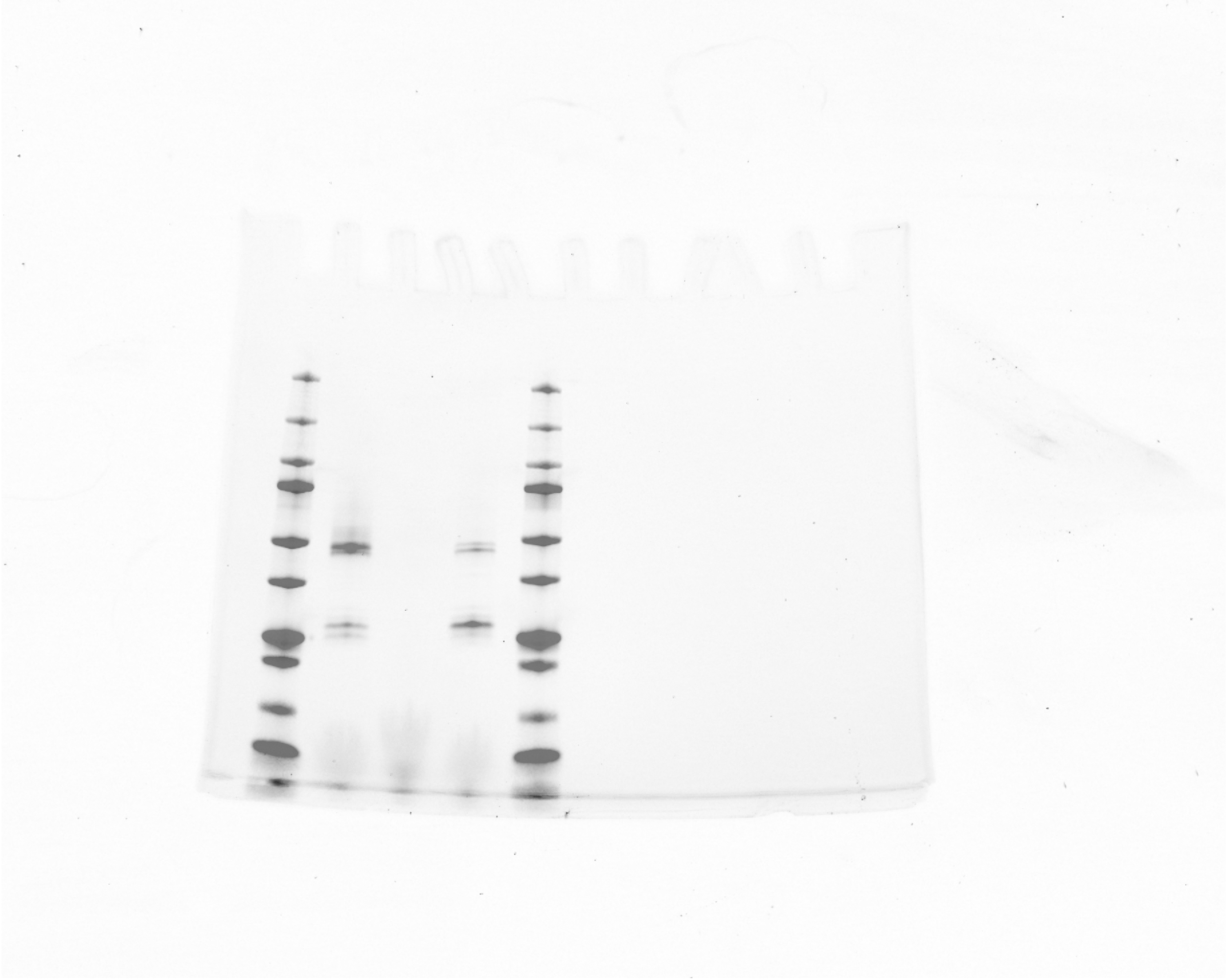

Supplement: Figure 4—figure supplement 1—source data 2. [file elife-106134-fig4-figsupp1-data2.zip › Figure-supplementary-4_source-data_raw/CALHM6_signal_gel2.png]

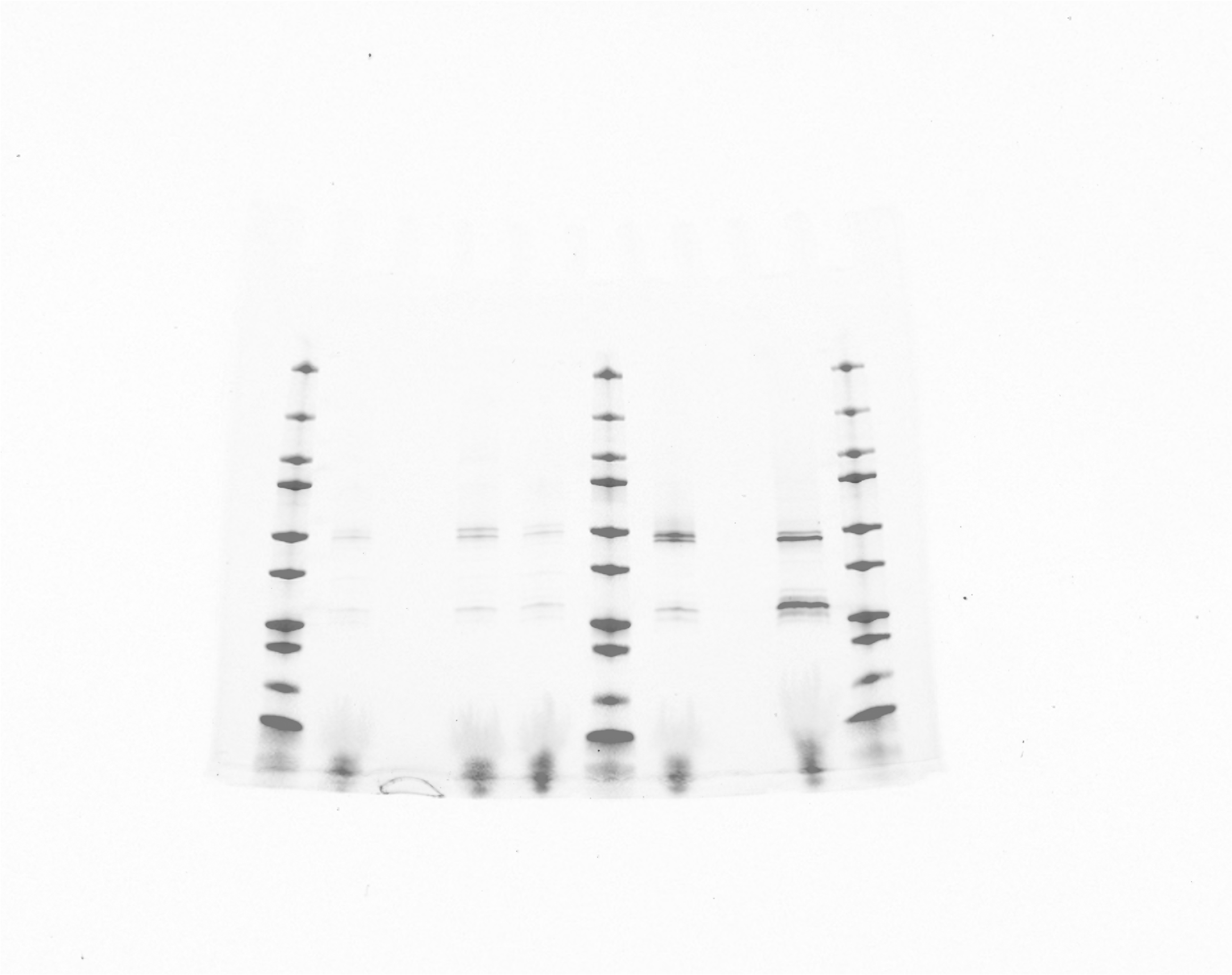

Supplement: Figure 4—figure supplement 1—source data 2. [file elife-106134-fig4-figsupp1-data2.zip › Figure-supplementary-4_source-data_raw/CALHM6_signal_gel3.png]

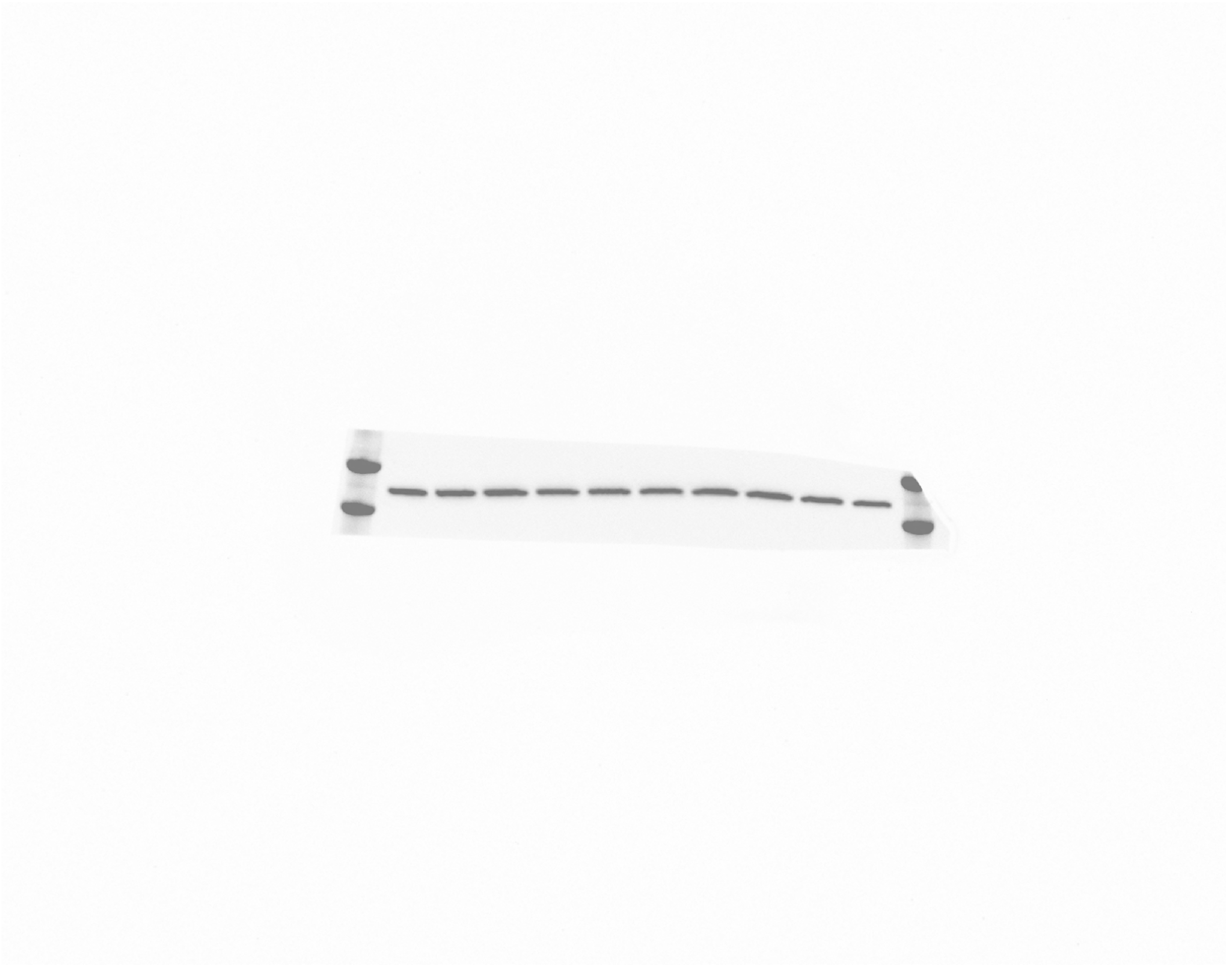

Supplement: Figure 4—figure supplement 1—source data 2. [file elife-106134-fig4-figsupp1-data2.zip › Figure-supplementary-4_source-data_raw/CALHM1_beta-actin_WB1.png]

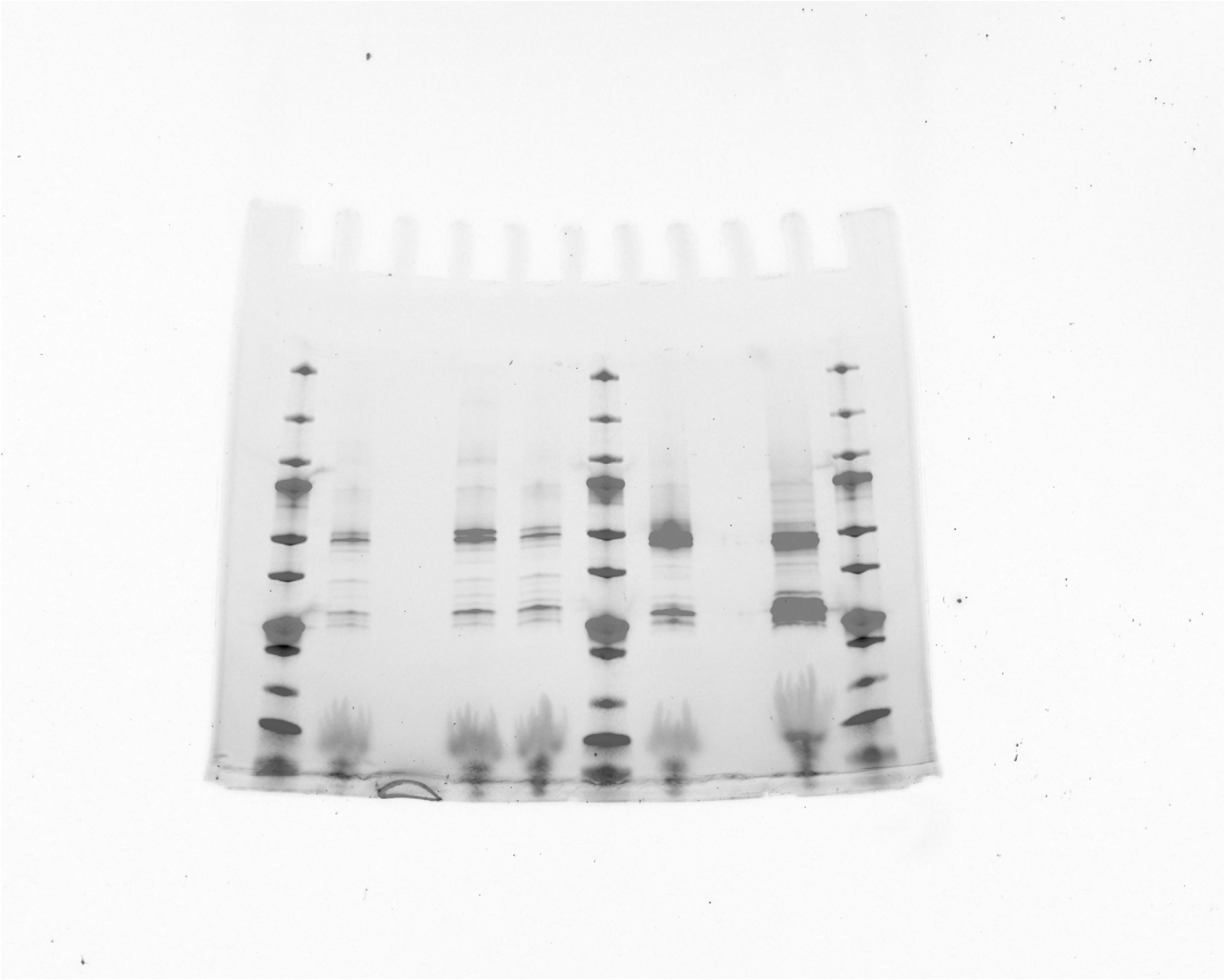

Supplement: Figure 4—figure supplement 1—source data 2. [file elife-106134-fig4-figsupp1-data2.zip › Figure-supplementary-4_source-data_raw/CALHM1_signal_gel2.png]

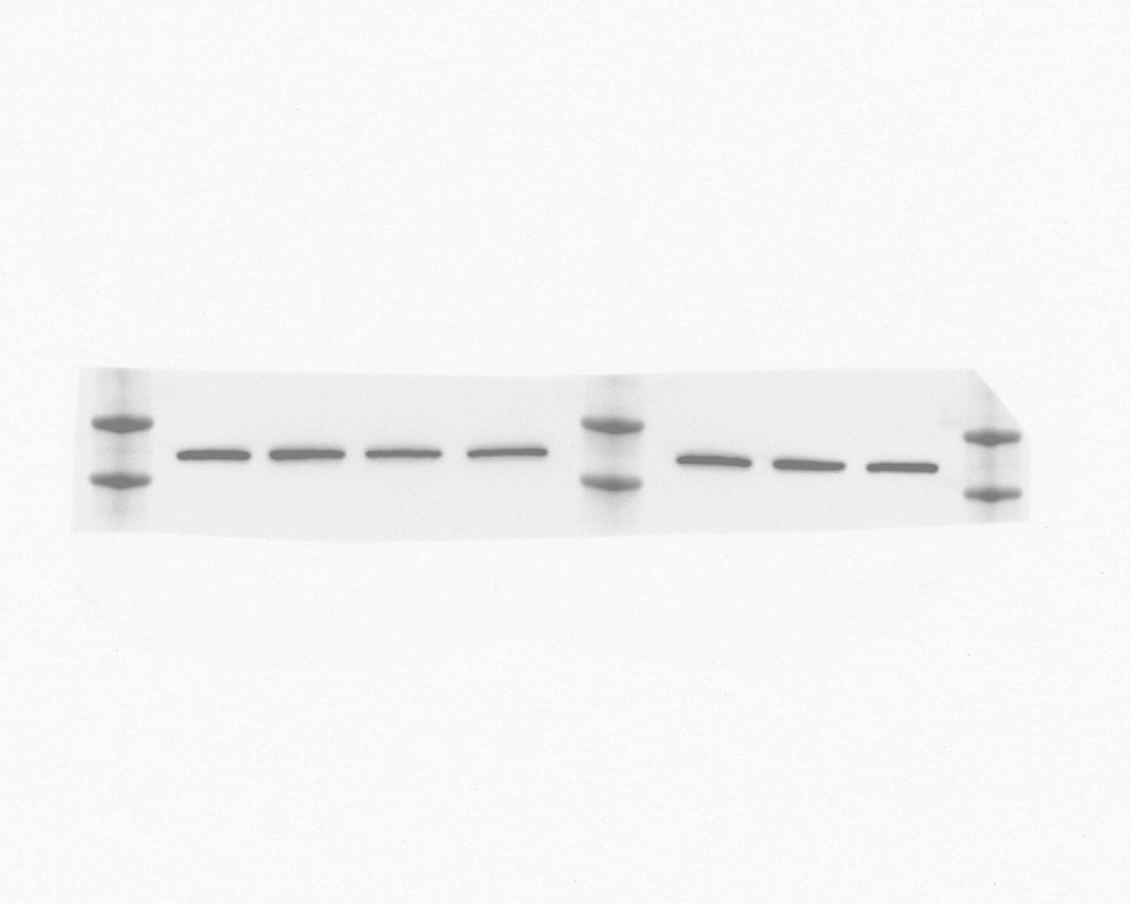

Supplement: Figure 4—figure supplement 1—source data 2. [file elife-106134-fig4-figsupp1-data2.zip › Figure-supplementary-4_source-data_raw/CALHM1_beta-actin_WB2.png]

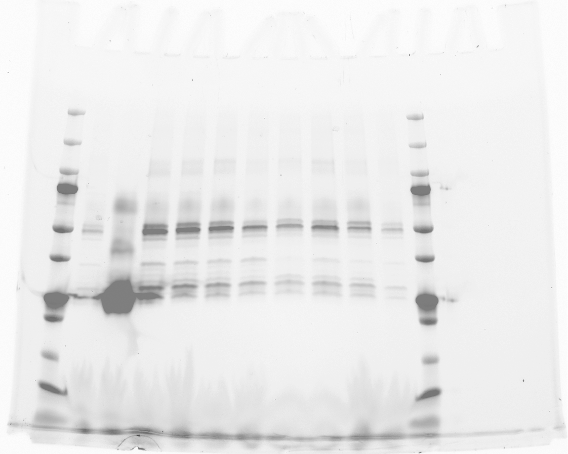

Supplement: Figure 4—figure supplement 1—source data 2. [file elife-106134-fig4-figsupp1-data2.zip › Figure-supplementary-4_source-data_raw/CALHM1_signal_gel1.png]

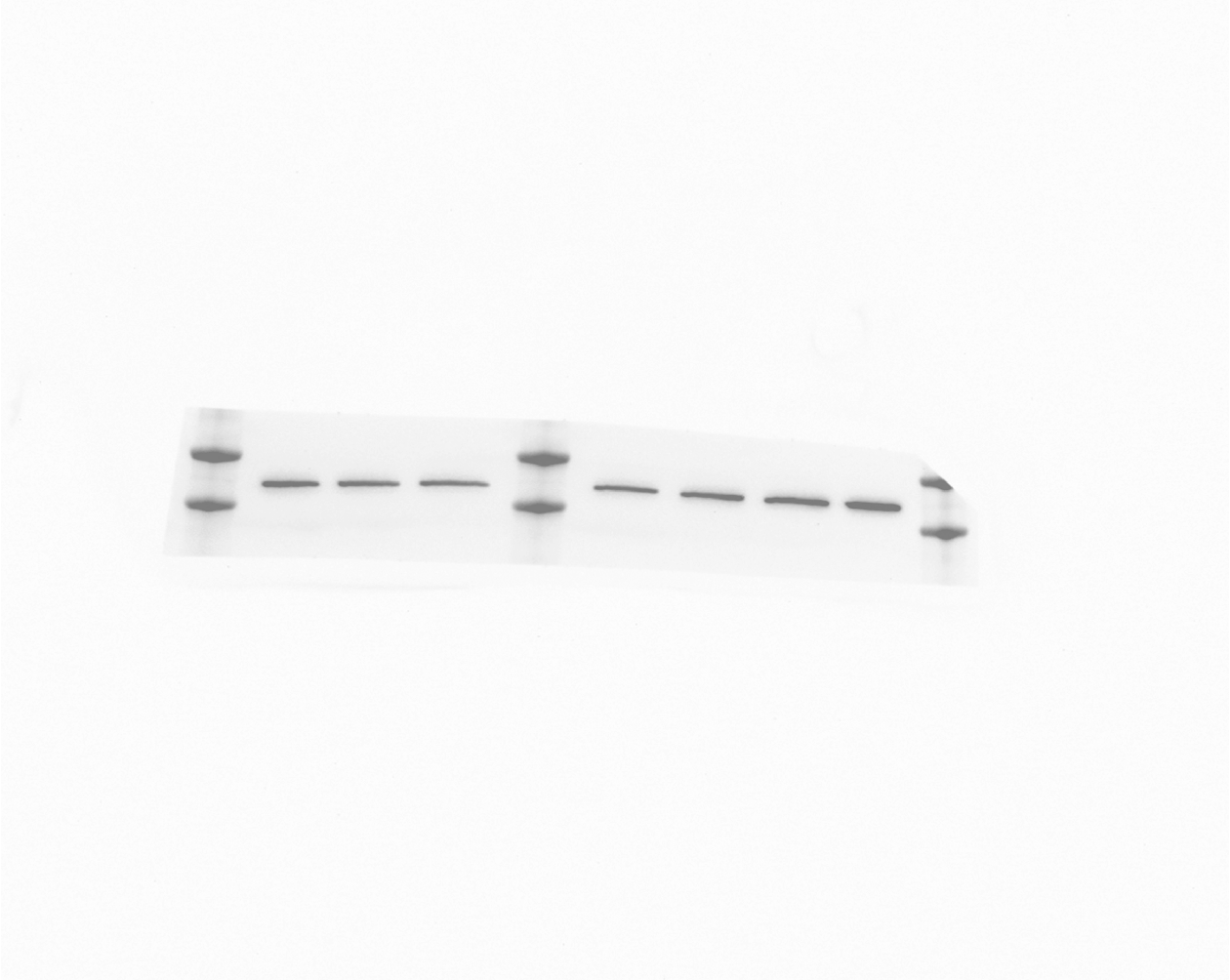

Supplement: Figure 4—figure supplement 1—source data 2. [file elife-106134-fig4-figsupp1-data2.zip › Figure-supplementary-4_source-data_raw/CALHM6_beta-actin_WB3.png]

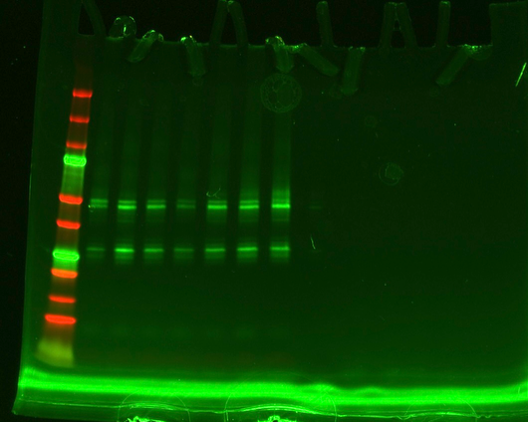

Supplement: Figure 4—figure supplement 1—source data 2. [file elife-106134-fig4-figsupp1-data2.zip › Figure-supplementary-4_source-data_raw/CALHM6_biotinylation_gel.png]

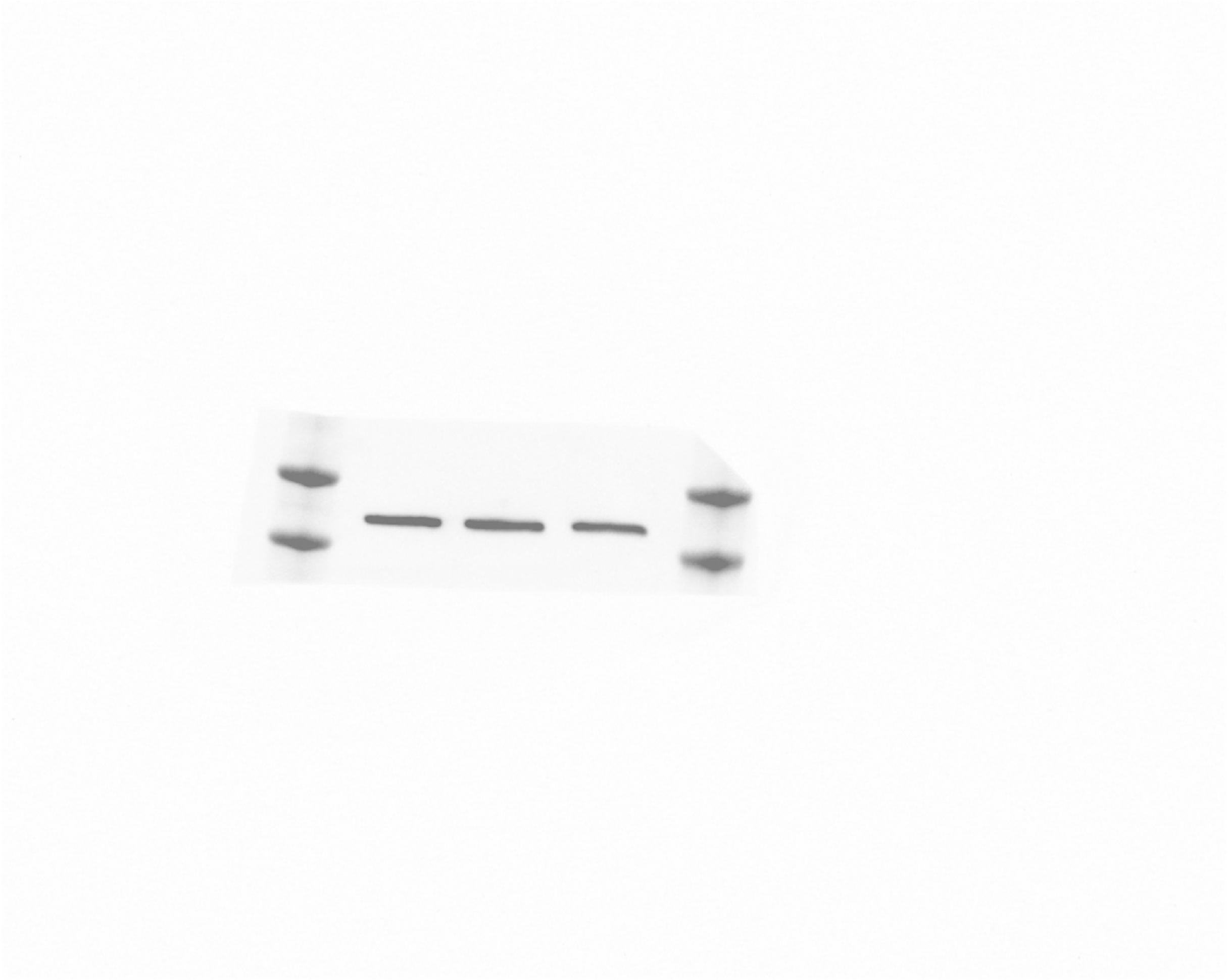

Supplement: Figure 4—figure supplement 1—source data 2. [file elife-106134-fig4-figsupp1-data2.zip › Figure-supplementary-4_source-data_raw/CALHM6_beta-actin_WB2.png]

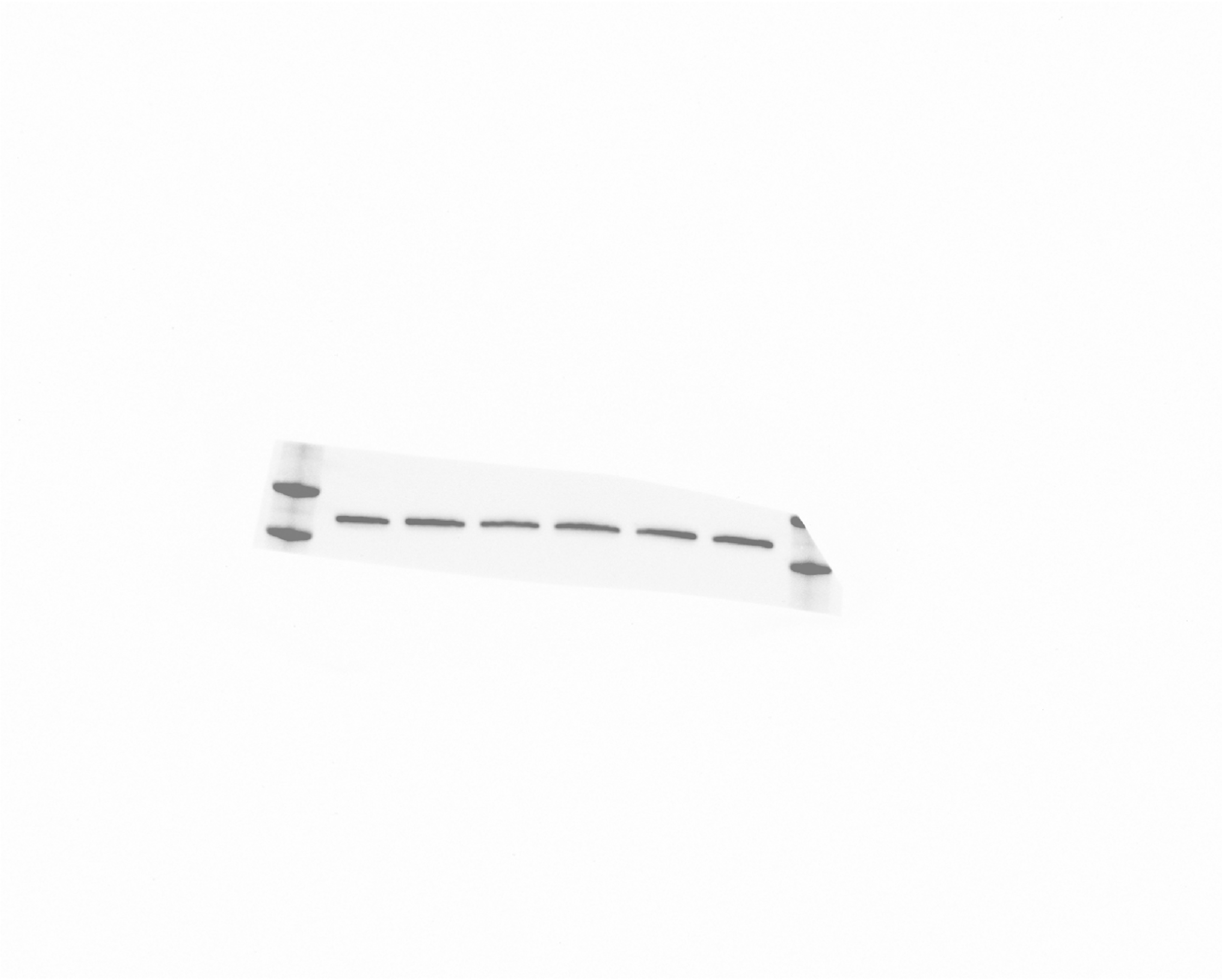

Supplement: Figure 4—figure supplement 1—source data 2. [file elife-106134-fig4-figsupp1-data2.zip › Figure-supplementary-4_source-data_raw/CALHM6_beta-actin_WB1.png]
